# Why can some implicit Theory of Mind tasks be replicated and others cannot? A test of mentalizing versus submentalizing accounts

**DOI:** 10.1371/journal.pone.0213772

**Published:** 2019-03-25

**Authors:** Louisa Kulke, Josefin Johannsen, Hannes Rakoczy

**Affiliations:** 1 University of Göttingen, Institute of Psychology, Göttingen, Germany; 2 Leibniz ScienceCampus Primate Cognition, Göttingen, Germany; SWPS University of Social Sciences and Humanities, POLAND

## Abstract

In the last 15 years, Theory of Mind research has been revolutionized by the development of new implicit tasks. Such tasks aim at tapping children’s and adults’ uninstructed, largely automatic mental state ascription, indicated in spontaneous looking behavior when observing agents who act on the basis of false beliefs. Studies with anticipatory looking, in particular, have suggested that basic ToM capacities operate from very early in life and remain in unconscious operation throughout the lifespan. Recently, however, systematic replication attempts of anticipatory looking measures have yielded a complex and puzzling mixture of successful, partial and non-replications. The present study aimed at shedding light on the question whether there is a system to this pattern. More specifically, in a set of three preregistered experiments, it was tested whether those conditions that could previously be replicated and those that could not differ in crucial conceptual respects such that the former do not strictly require ToM whereas the latter do. This was tested by the implementation of novel control conditions. The results were complex. There was generally no unambiguous evidence for reliable spontaneous ToM and no effect of the number of passed familiarization trials. Neither was there any unambiguous evidence that the previous mixed patterns of (non-)replications could be explained (away) by the sub-mentalizing account tested in the new control conditions. The empirical situation remains puzzling, and the question whether there is some such thing as implicit and spontaneous ToM remains to be clarified.

## Introduction

Theory of Mind, the ability to ascribe beliefs, desires and other mental states to others and ourselves, has traditionally been considered to develop between the ages of 3 and 5 years, depending on cultural and linguistic experience and central cognitive resources [1,2]. New findings from the last 15 years, however, have fundamentally challenged this picture and revolutionized the field. These findings stem from implicit Theory of Mind (ToM) tasks that operate without direct verbal measures or instruction. The evidence from studies with such tasks indicates that basic forms of ToM develop very early and are to be found even in infants. Across the lifespan, they may continue to operate in largely automatic and unconscious ways (see [3] for a review). Implicit tasks include *interactive behavioral tasks* [4,5], *violation of expectation* looking time paradigms [6,7], and *anticipatory looking* (AL) measures (e.g. [8,9–13]).

The most comprehensive body of evidence comes from AL false belief (FB) tasks. Such tasks build on the litmus test of explicit ToM, the standard explicit change-of-location FB task [1]: an object is transferred in the absence of a protagonist, and the subsequent test question is where the protagonist will look for the object. Rather than explicitly asking the participant where the protagonist will search, implicit AL tasks tap participants’ spontaneous looking behavior: when the agent returns to the scene, will participants anticipate her to search in accordance with her belief and look towards the corresponding location [13]? AL has been used from infancy to adulthood, across species, and across neurotypical and atypical populations. Results suggest that implicit ToM emerges early and remains in operation across the human lifespan [8,10], might even be present in non-human primates [14], and may differ in subtle yet crucial ways between neurotypical and autistic adults (see e.g. [9,10]).

Ambitious theoretical accounts have been developed on the basis of these findings, including nativists views [15–18] and two-systems accounts [19,20]. Both kinds of theories take the AL findings to suggest that there are early-developing, automatic and perhaps modular forms of ToM. In contrast to accounts assuming that implicit ToM exists, skeptical accounts doubt that the implicit findings describe a real form of ToM like the traditional explicit tasks. Instead, submentalising accounts (e.g., [21]) suggest that early tasks measure simple sensory and attentional processes (e.g., attraction of gaze to specific locations, without any requirement to predict an agent’s behavior or mental state at all) or use of behavioral rules (i.e. predicting a person’s future behavior based on a standard rule such as "a person will look for an object where he/she last saw it" that do not involve any ascription of mental states (e.g. [22]). Current disputes mainly center on the question which account best explains the existing set of original findings from AL tasks.

From an empirical point of view, however, it has recently become less and less clear how robust and reliable these original findings are in the first place. First attempts at independent, systematic and large-scale replication of these findings have yielded a complex mixture of partial and unsuccessful replications. Two different kinds of AL FB tasks have been used in these replication studies: *Transfer tasks* are implicit versions of standard Wimmer & Perner change-of-location tasks [8,11]. A protagonist puts an object in box 1. The object is then transferred in the presence (true belief (TB) condition) or absence (FB condition) of the protagonist. Finally the protagonist sets out to get his object, and the dependent measure is participants’ anticipatory looking: Do they look in anticipation to where the agent believes the object to be, i.e., to box 1 in the FB condition and to box 2 in the TB condition?

In *Removal tasks*, in contrast, the target object is removed from the scene rather than transferred in the protagonist’s absence [9,10]. Thus, in the crucial anticipation phase, firstly there is not the same conflict as in transfer tasks between the object’s real location and the location at which the protagonist believes it to be. Secondly, the salient location of the object cannot interfere with participants’ gaze patterns, as it is not visible. Removal tasks come in two FB condition. In both conditions, the target object is first placed in box 1, then transferred to box 2 and then removed from the scene. In FB1, the protagonist witnesses steps 1+2, but not step 3. The protagonist thus falsely believes that the object is still located in box 2, which is also the last location the object was in before removal. In FB2, the protagonist witnesses step 1, but not steps 2 and 3. The protagonist thus has the false belief that the object is in box 1.

The pattern that emerges from a growing number of large-scale replication studies, many of them direct replications with the exact same stimuli and procedures, yet with much larger samples sizes than original studies, is the following: In both children and adults, non-verbal *transfer tasks* could, so far, not be robustly replicated independently [23,24]. Results of the replication studies revealed that participants mostly looked in anticipation to box 2 (the object’s real location) both in TB and FB conditions. Regarding *removal tasks*, some studies with children and adults could not replicate any original findings [25]. Most studies, however, successfully replicated only FB1 while failing to replicate FB2 [23–30].

The central question is whether some systematic causes can be identified underlying this complex pattern of (non-)replications. May there be an underlying conceptual difference between those tasks that do and those that do not replicate that explains why this is the case? A closer look at the different conditions reveals one obvious candidate: the only condition that proved robustly replicable so far, FB1 of the removal tasks, is the only FB condition in which there is a basic confound: the correct belief-based location (box 2) is also the object’s last location before removal. Perhaps, thus, participants do not look in anticipation based on the belief ascribed to the agent, but simply look in anticipation based on the low-level (sub-mentalizing) rule that people typically search where an object last was (alternative explanation, previously suggested e.g. by [24,31]). In their original study, Southgate et al. (2007) implemented the FB2 condition to control if participants look at the last object location, which competes with the belief-congruent location in this condition. Unfortunately. The FB2 conditions proved to be least replicable in previous research, with gaze patterns in replications indicating object tracking rather than belief tracking [23,24,32].

Therefore, the main rationale of the present study was to investigate the possibility of object tracking, firstly by testing, again, the replicability of FB1 and FB2. Secondly, the crucial novel step was that we devised a new true belief control condition (TB1) that was identical to the FB1 condition, with one fundamental exception: the protagonist witnessed all relevant steps (object is put in box 1, is then transferred to box 2 and finally removed from the scene). This new condition helps to disambiguate previous replication patterns, since the different possibilities left open so far create diverging predictions for TB1: If participants simply look to the object’s last location, they should reveal the same looking pattern as in FB1 and mostly look towards box 2. If, however, their looking behavior reflects belief-based anticipatory looking, participants should not look systematically towards either location, since they know that the protagonist knows that the object is no longer in the scene.

In addition, two complementary aims were to probe whether replicability of AL tasks could be improved: First, may more controlled stimulus materials affect gaze patterns? This question aimed at reducing the effect of potential confounds with the turning direction of the actress.

Second, AL tasks in both original and replication studies, suffer from massive exclusion rates. Participants watch 2–4 familiarization trials in which the protagonist searches for her object and in which there is no question of false belief yet. Only participants who spontaneously show anticipatory looking in these baseline trials are included in the main analysis of the test trials, resulting in intolerable exclusion rates between 30 and 50%. In Study 3, we aimed to alleviate this unfortunately high exclusion rate by implementing a novel, more fine-grained familiarization procedure. Since original AL findings suggest stable patterns of anticipatory looking across the lifespan, both adults (Studies 1 and 3) and children (Study 2) were tested.

## Studies 1 and 2

### Method

#### Participants

Study 1 was preregistered with the Open Science Framework (https://osf.io/d47zk/). Participant numbers were pre-determined as a minimum of 25 per condition based on the original studies, leading to a total number of 75 participants. For Study 1, 135 healthy adult participants were tested in total to reach the pre-determined number of 75 included participants (Mean age = 23.68 years, SD = 3.40, 63 male). They received sweets in return for participation. Study 2 used identical stimuli and procedures as Study 1, but tested children between 4.5 and 5.5 years. 116 children were tested (Mean age = 59.78 months, SD = 4.12, 61 male) to reach the pre-determined number of 75 included participants. Participants were excluded if they did not attend to the correct window during the last familiarization. The study was approved by the University of Göttingen ethics committee (Ref. number: 143b). All investigation have been conducted according to the principles expressed in the Declaration of Helsinki. Written informed consent was obtained from adult participants and parents of under-age participants. Additional verbal assent was obtained from children.

#### Apparatus and materials

**Fig 1:**
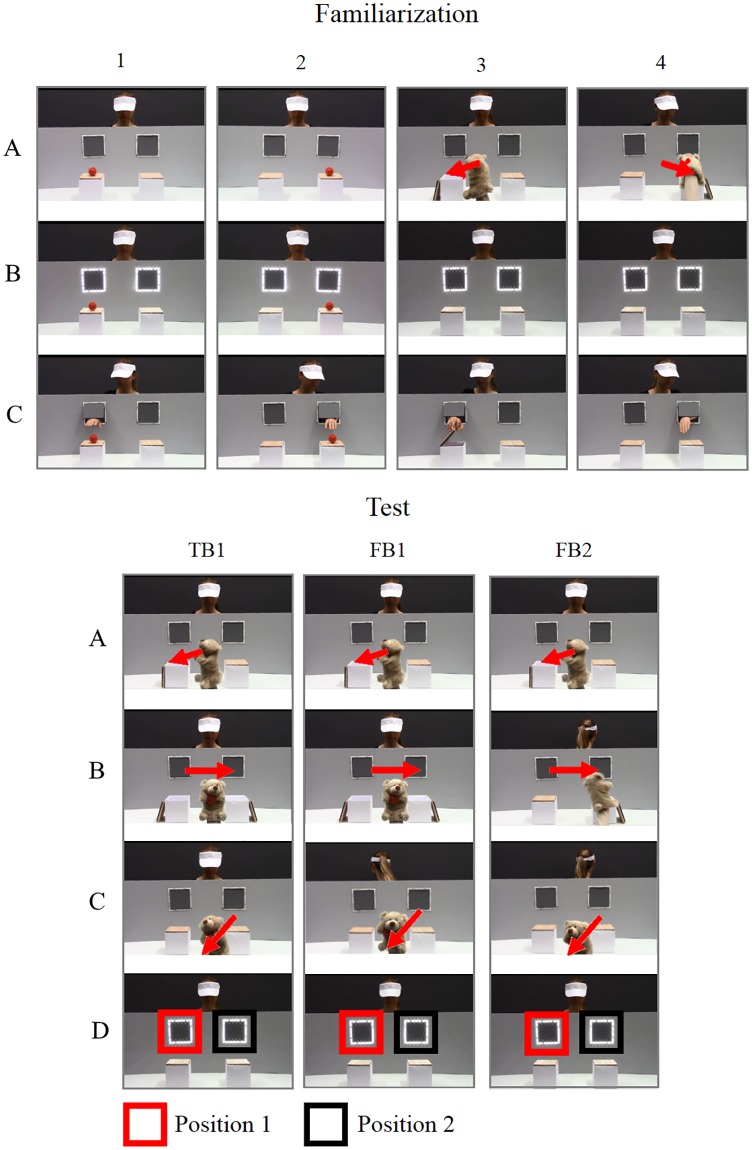
Schematic display of the events during familiarization trials 1 to 4 (top), and test trials FB1, FB2 and TB1 trials (bottom).

The stimuli were based on the study by Southgate et al. [9,10]. The setting was as similar as possible to the original videos, with a hand-puppet and an actress interacting (Fig 1). Like in the original stimuli, the actress was wearing a visor and was separated from the puppet by an occluder with two windows, one on the left and one on the right side of the actress. In front of every window there was a box. Each condition started with two familiarization trials in which the actress was sitting behind the occluder, a ball was lying on one of the boxes (one trial left, one trial right), a chime sounded and the windows both lit up and the actress subsequently reached for the ball after a delay of 1.75 sec. In the subsequent two familiarization trials a bear appeared from the central bottom of the screen, holding the ball. It placed the ball into one of the two boxes (one trial left, one trial right) and closed the lid. It then disappeared at the center. The chime sounded again, followed by the actress reaching through the respective window for the ball. Videos were edited using Adobe Premiere Elements 10 Software to ensure that the delay between the chime sound and the reaching was the same in all conditions. In the test trial, the bear appeared and placed the ball in the left box (position 1). It then moved the ball to the other box (position 2). Finally, the bear removed the ball from the scene. During the procedure, a phone started to ring and the actress turned away either only during the removal of the ball (FB1 condition), meaning that the actress last saw the ball in box 2, or during the moving and the removal of the object, meaning that the actress last saw the ball in box 1 (FB2 condition). In both trials the actress then turned back, a chime sounded and the windows lit up. In addition to the original FB1 and FB2 condition, we introduced a novel true belief (TB1) condition, in which the actress did not turn away but witnessed the moving and the removal of the ball, followed by the chime sounding and the windows lighting up. Both the actress and the viewer hence last saw the ball in box 2 but know that it has been removed. Note that, based on the original study by Southgate et al., the ball was always placed in the left box first in the test trials; hence, the final location of the ball was only counterbalanced in the familiarization, but not in the test trials. The stimulus videos are provided at the DOI 10.17605/OSF.IO/EF629. Table 1 shows corresponding predictions of AL patterns depending on whether participants adopt a where-object-was-last rule or belief-based reasoning, as function of condition. *X* marks the predicted search location and *n*.*a*. marks cases in which no prediction can be derived from a certain search strategy.

**Table 1:** Predicted AL patterns depending on adopted search strategy (where-object-was-last rule or belief-based reasoning) as a function of condition (TB1, FB1 or FB2).

	TB1		FB1		FB2	
	box 1	box 2	box 1	box 2	box 1	box 2
Sub-mentalizing (where-object-was-last rule)		X		X		X
Mentalizing (belief-based reasoning)	n.a.	n.a.		X	X	

#### Equipment

An SMI RED250mobile eye tracker recorded gaze positions at a rate of 60 Hz. Before recording started, participants completed a standard 5 point calibration and validation routine. Movies were controlled in the SMI Experiment Center (version 3.6.54) and presented on a separate DELL P2211H 21.5´´ LCD screen (1920 x 1080 pixel). Gaze information was saved for offline analysis, which was conducted using BeGaze Software (version 3.6.54), RStudio and IBM SPSS Statistics (version 25).

#### Procedure

Participants signed an informed consent form and were seated in front of the eye-tracker. After the calibration procedure they saw four familiarization trials and one of the three test trials.

#### Eye tracking analysis

Participants were excluded if they did not attend to the correct window during the last ‘ball-in-box’ familiarization, in line with the original study. However, there was no measurement of AL in the last ‘ball-on-box’ familiarization because it was assumed that participants’ gaze would be attracted to the appropriate window in those types of familiarizations.

As in the original study, first saccades and looking time to an AOI (referred to as position 1 and position 2) consisting of the respective window above box 1 and box 2, measured through differential looking scores (DLS) were computed as outcome variables. In the FB1 and FB2 condition, the DLS was calculated as the difference in looking time to the incorrect side AOI from the correct side AOI divided by the sum of looking times to both sides:

$$DLS_{belief-based}=\frac{\text{correct}\;\text{position}-\text{incorrect}\;\text{position}}{\text{correct}\;\text{position}+\text{incorrect}\;\text{position}}$$

This DLS can vary between -1 and 1, with 1 meaning perfect performance in terms of belief-congruent anticipatory looking, -1 meaning that participants only looked towards the belief-incongruent location, and a score of zero indicating that participants did not look longer to either the belief-congruent or belief-incongruent side.

In order to control for object tracking as an alternative explanation, i.e. participants simply looking to the last object location, FB1 and TB1 were compared. A DLS was calculated accordingly as the difference in looking time to the last object location at position 2 from the last empty location at position 1 divided by the sum of looking times to both sides:

$$DLS_{where-object-last-was}=\frac{\text{position}\;\text{2}-\text{position}\;\text{1}}{\text{position}\;\text{2}+\text{position}\;\text{1}}$$

This DLS can vary between -1 and 1, with 1 meaning an increased looking duration towards position 2, which is equal to the last location of the object, -1 meaning that participants showed an increased looking duration towards position 1, the last empty location, and a score of zero indicating that participants did not look longer to either the last object or last empty location. In case of FB1, position 2 is both the belief-congruent and last object location; this DLS_where-object-last-was_ is therefore equivalent to DLS_belief-based_.

Effects on the direction of first saccades (binary variable) were determined using binomial tests. DLS was compared with zero using one-sample t-tests and between conditions using independent samples t-test.

Follow up Bayesian analyses were conducted using the “BayesFactor” Package [33] in R [34] using Cauchy priors based on Liang et al. [35]. As preregistered, Bayes Factors were computed whenever tests were non-significant based on an alpha level of .05.

### Results

**Fig 2:**
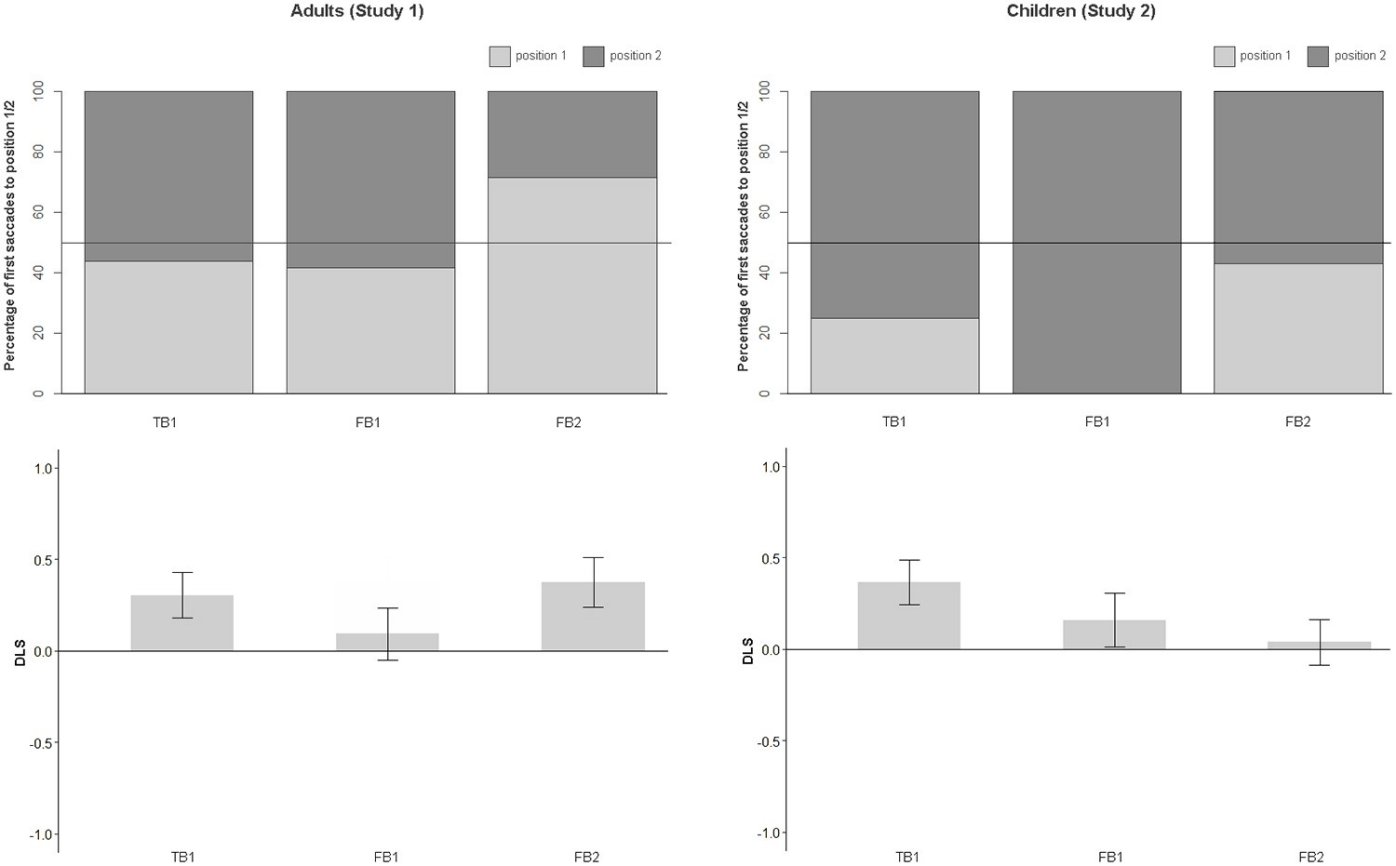
Percentage of first saccades (top) and DLS (bottom) to both positions in adults (left panels) and children (right panels).

Full datasets are provided at the DOI 10.17605/OSF.IO/EF629. The percentage of first saccades and the proportion of looking time to position 1 and position 2 are depicted in Fig 2 for both age groups. Additional preregistered analyses are reported in S1 File.

#### Adults

**First saccade**. As only one first saccade was measured per participant, binomial tests rather than the planned mixed logistic regressions were computed. Binomial tests showed that the first saccade was not significantly more often directed to either position in the FB1 condition (7 out of 12, i.e. 58% to position 2, *p* = .774, *BF*_*10*_ = 0.643), indicating no preference for the last object or belief-congruent location, the same applies to the FB2 condition (5 out of 7, i.e. 71% to position 1, *p* = .453, *BF*_*10*_ = 0.965), indicating no preference for the belief congruent location, and the TB1 condition (9 out of 16, i.e. 56% to position 2, *p* = .804, *BF*_*10*_ = 0.571), indicating no preference for the last object location. The remaining participants (13 in FB1, 18 in FB2, 9 in TB1) showed no eye-movement to either the box 1 or box 2 location. A chi-square test showed no significant differences in correct saccades between conditions, *chi*^*2*^ = 0.303, *p* = .860, *BF*_*10*_ = 0.158.

**DLS**. One-sample t-tests were used to investigate whether the differential looking score differs from zero in each condition. DLS_belief-based_ did not significantly differ from zero in the FB1 condition (*M*(25) = 0.094, *SD* = 0.678, 95% CI [-0.19, 0.37]), *t*(24) = 0.696, *p* = .493, *d* = 0.284, *BF*_*10*_ = 0.263, but was significantly positive in the FB2 condition (*M*(25) = 0.376, *SD* = 0.635, 95% CI [0.11, 0.64]), *t*(24) = 2.959, *p* = .007, *d* = 1.208. The DLS_where-object-last-was_ in the TB1 condition was also significantly different from zero, (*M*(25) = 0.304, *SD* = 0.593, 95% CI [0.06, 0.55]), *t*(24) = 2.564, *p* = .017, *d* = 1.047.

An independent samples t-test showed no significant difference of DLS_belief-based_ between FB1 and FB2, *t*(48) = -1.515, *p* = .136, *d* = -0.437, *BF*_*10*_ = 0.718, indicating comparable performance in terms of belief-congruency. The analysis revealed no significant difference of DLS_where-object-last-was_ between FB1 and TB1, *t*(48) = -1.164, *p* = .250, *d* = -0.336, *BF*_*10*_ = 0.492, indicating comparable patterns of looking durations in both conditions.

#### Children

**First saccade**. Binomial tests showed that the first saccade was not significantly more often directed to either position in the FB1 condition (4 out of 4, i.e. 100% to position 2, *p* = .125, *BF*_*1*0_ = 1.900), indicating no preference for the last object or belief-congruent location, the same applies to the FB2 condition (3 out of 7, i.e. 43% to position 1, *p* = 1, *BF*_*1*0_ = 0.701), indicating no preference for the belief-congruent location, and the TB1 condition (6 out of 8, i.e. 75% to position 2, *p* = .289, *BF*_*1*0_ = 1.179), indicating no preference for the last object location. A chi-square test showed no significant differences in correct saccades between conditions, *chi*^*2*^ = 2.423, *p* = .298, *BF*_*10*_ = 0.989.

**DLS**. One-sample t-tests were used to investigate whether the differential looking score differs from zero in each condition. DLS_belief-based_ did not significantly differ from zero in the FB1 condition (*M*(25) = 0.160, *SD* = 0.630, 95% CI [-0.16, 0.42]), *t*(24) = 1.266, *p* = .218, *d* = 0.517, *BF*_*10*_ = 0.430, nor in the FB2 condition (*M*(25) = 0.038, *SD* = 0.588, 95% CI [-0.21, 0.28]), *t*(24) = 0.324, *p* = .748, *d* = 0.132, *BF*_*10*_ = 0.221, but DLS_where-object-last-was_ was significantly positive in the TB1 condition (*M*(25) = 0.368, *SD* = 0.585, 95% CI [0.13, 0.61]), *t*(24) = 3.141, *p* = .004, *d* = 1.282, *BF*_*10*_ = 9.552.

Mixed linear models with participant ID as random effects and Condition as fixed factor showed no significant effect of condition, *F*(2, 72) = 1.919, *p* = .154, *BF*_*10*_(Condition+ParticipantIDvsSub) = 0.284. An independent samples t-test showed no significant difference of DLS_belief-based_ between FB1 and FB2, *t*(48) = 0.704, *p* = .485, *d* = 0.203, *BF*_*10*_ = 0.346, indicating comparable performance in terms of belief-congruency. The analysis revealed no significant difference of DLS_where-object-last-was_ between FB1 and TB1, *t*(48) = -1.211, *p* = .232, *d* = -0.350, *BF*_*10*_ = 0.405, indicating comparable looking durations towards position 1 and 2.

#### Exclusion rates

Note that 60 out of 135 (44.4%) of participants in Study 1 and 41 out of 157 (35.3%) of participants in Study 2 were excluded based on the original criteria. This exclusion rate is somewhat higher than in the original studies. Therefore, Study 3 aimed to modulate the familiarization procedure and further analyze the exclusion rate.

### Discussion

The main rationale of Studies 1–2 was to test for a low-level alternative to belief-tracking (i.e. tracking the last location of the target object) as an explanation for replicable anticipatory looking patterns in AL FB tasks. To this end, a new control condition was devised (TB1) that was identical to FB1 with the sole exception that the protagonist witnessed all crucial events, therefore knows that the object has disappeared and thus has no reason to approach any of the two boxes in search of the object. If participants show anticipatory looking just like in FB1 nonetheless, this would suggest that their gaze reflects tracking of the object’s last location rather than the agent’s belief.

The results of the present study were complex: First, there was indeed some evidence for low-level object tracking in TB1, both children and adults showed significantly longer looking at the previous ball location (i.e. position 2) than expected by chance. Note, however, gaze patterns in the other conditions do not always reflect object tracking. Second, children showed no gaze behavior indicating belief tracking in either FB1 or FB2, so that the study failed to replicate the original findings [9,10]. Third, adults showed no belief tracking in the FB1 but did so in the FB2 condition. This finding is very surprising given that the FB1 condition is less complex, since it involves no location change in the protagonist’s absence and the last ball location is thus identical to the belief-congruent location.

Why did participants perform more in line with belief-tracking in FB2 nonetheless? Here, we can only speculate. At a closer look, in the original and therefore also in the current study, the actress turns towards the belief-congruent location at the end of the trial to reengage with the scene. This turn might be cueing the participant to look towards the side the actress turns to, and may do so in particularly pronounced ways in the FB2 condition in the current stimulus material. This might have affected the gaze pattern more strongly and led to significantly more looking towards the turn-congruent, i.e. belief congruent location. Previous research [24] identified cueing as an alternative explanation for a different false belief paradigm by Low and Watts [12]. This alternative explanation could also apply to Studies 1 and 2 of the current paper, as well as the original studies by Southgate et al. Study 3 was therefore designed to exclude the possibility of a cueing effect by avoiding any turn of the actress and instead letting her enter the scene centrally. If human actors are filmed for stimulus creation, their behavior and movements will never be identical in all conditions, as even small muscle movements may be visible and affect gaze patterns of the viewer. Therefore, in Study 3, animated videos were used to ensure that no cueing was possible due to subliminal behavior of actors.

Studies 1–2, like many previous AL studies, also suffered from high exclusion rates: more than 40% of participants in the current study had to be excluded from the main analyses because they did not pass the inclusion criteria used in the original studies (they failed to correctly anticipate the actress’s behavior in the last ball-in-box familiarization). A complementary aim of Study 3 was to improve the familiarization procedure and thus reduce exclusion rates by implementing a more fine-grained familiarization and inclusion criterion. In most original paradigms (e.g., [9,10]), only the performance in the final out of two or four familiarization trials was used to infer participants’ task comprehension and sufficient task engagement. However, by using a discrete inclusion criterion based on a single trial the risk of participants’ passing/failing the criterion merely by chance is quite high. Therefore, to reduce the risk of random inclusion of participants we used the ratio of passed trials rather than performance in a single trial in study 3.

## Study 3

### Method

#### Participants

The study was pre-registered with the Open Science Framework (https://osf.io/gfjs5/). Participant numbers were pre-determined as a minimum of 25 per condition based on the original studies, leading to a total number of 75 participants. 157 healthy adult participants were tested in total to reach the pre-determined number of 75 included participants (Mean age = 23.83 years, SD = 3.22; 39 male, 35 female and 1 without gender information). They received sweets in return for participation. Participants were excluded if they did not attend to the correct position in at least two out of the last three familiarization trials. The study was approved by the University of Göttingen ethics committee (Ref. number: 143b). All investigation have been conducted according to the principles expressed in the Declaration of Helsinki. Written informed consent was obtained from all participants.

#### Apparatus and materials

**Fig 3:**
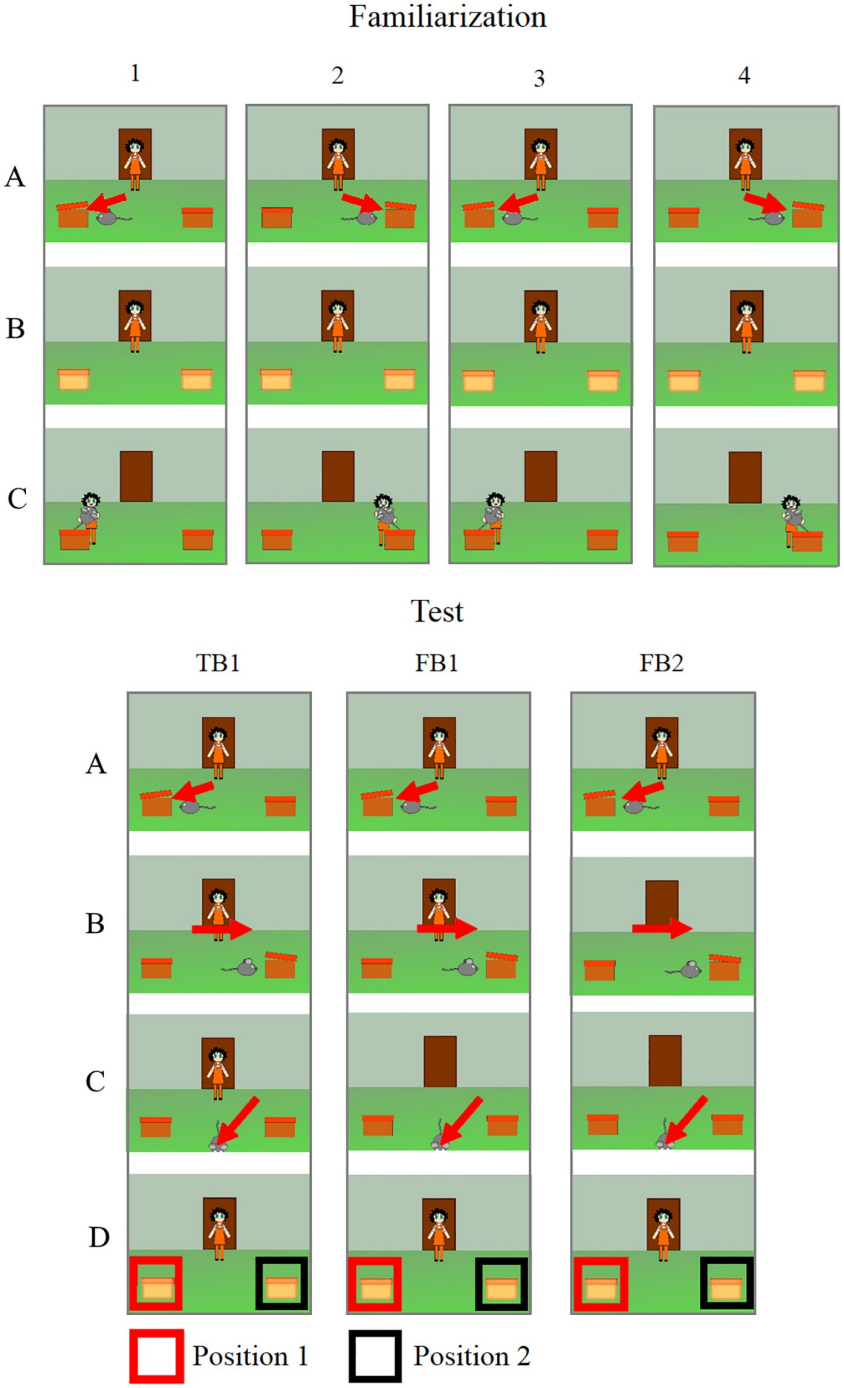
Schematic display of the events during the 4 subsequent familiarization trials (1–4, top), and the test trials FB1, FB2 and TB1 trials (bottom).

The stimuli were based on the study by Southgate et al. [9,10]. While the basic structure of the original videos was kept, the current stimuli differed regarding several aspects from the original material: (1.) A mouse was used as an object and was moving by itself instead of being hidden by another agent. (2.) While the object changed locations the agent left the room through a central door instead of being distracted. (3.) There were no windows included, only two boxes, which were illuminated instead as a cue for the following search behavior. (4.) Instead of real life recordings, animated videos were used. Videos were edited using Vegas Movie Studio HD Platinum (version 11.0). Events shared by different videos (e.g. hiding of the object) were always composed of the same animated sequences and therefore allowed for exact timing control between conditions. Each condition started with 4 familiarization trials, in which a human agent entered a room through a door centered in the background of the scene, thereafter a mouse entered from the central bottom of the screen and hid itself in one of two boxes (right or left) in front of the agent. A chime sounded and the boxes were lit up and the agent retrieved the mouse after a delay of 2.7 sec. In the test trial, the mouse firstly hid itself in one of the boxes (position 1) and thereafter changed locations into the remaining box (position 2), before leaving the scene altogether (Fig 3). In the FB1 condition, the mouse changed locations in presence of the agent. Subsequently the agent left the scene through the door. While the agent was gone, the mouse left the scene, afterwards the agent re-entered the scene. In the FB2 condition both location change and leaving the scene happened in absence of the agent. In addition to the original FB1 and FB2 condition, a TB1 condition was introduced (comparable to Study 1), in which the agent left and re-entered the scene only after witnessing both location change and leaving of the mouse. Re-entering of the scene was followed by the chime sounding and the boxes lighting up, the agent did not move for the remaining 6 sec. of the videos. The videos are provided at the OSF DOI 10.17605/OSF.IO/EF629.

#### Equipment

An SMI RED250mobile eye tracker recorded gaze boxes at a rate of 60 Hz. Before recording started, participants completed a standard 5 point calibration and validation routine. Movies were controlled in the SMI Experiment Center (version 3.6.54) and presented on the accompanying laptop screen (1920 x 1080 pixel). Gaze information was saved for offline analysis, which was conducted using BeGaze Software (version 3.6.54) and RStudio (version 1.0.136).

#### Procedure

Participants signed an informed consent form and were seated in front of the eye-tracker. After the calibration procedure they saw four familiarization trials and one of the three test trials. It was approximately counterbalanced between participants whether object/agent movements in the test trial were directed to the left or right side of the screen.

#### Eye tracking analysis

As in the original study, first saccades and looking time, measured through a differential looking scores (DLS) were computed as outcome variables.

In order to analyze effects on first saccades, binomial tests were used to investigate whether first saccades were significantly more often directed towards the belief-congruent location within each condition and Fisher’s exact tests were used to investigate whether this effect differed between conditions (FB1, FB2 and TB1). Taking into account that both study 1 and 2 often showed small individual sample sizes to compare, we preregistered Fisher’s exact tests instead of a chi-squared test for this part of the analysis in study 3. One-Sample t-tests were used to investigate whether the DLS differed from 0 within each condition. Independent samples t-tests were conducted to compare the FB1, FB2 and TB1 conditions.

The number of passed familiarization trials was measured both to check if the above mentioned familiarization criterion was met and to investigate potential effects on the DLS in FB1 and FB2 conditions. A linear regression was conducted including all participants independent of their performance during familiarization. To determine the potential effect of familiarization procedure on dropouts, the dropout rate was calculated. Fisher’s exact test was used to investigate whether the dropout rate differed between the current study and the original study by Southgate et al. (2007). Standard p-values of 0.05 served as a cut-off and two-tailed tests were used. In case of null effects, tests were followed up with Bayesian analyses.

### Results

**Fig 4:**
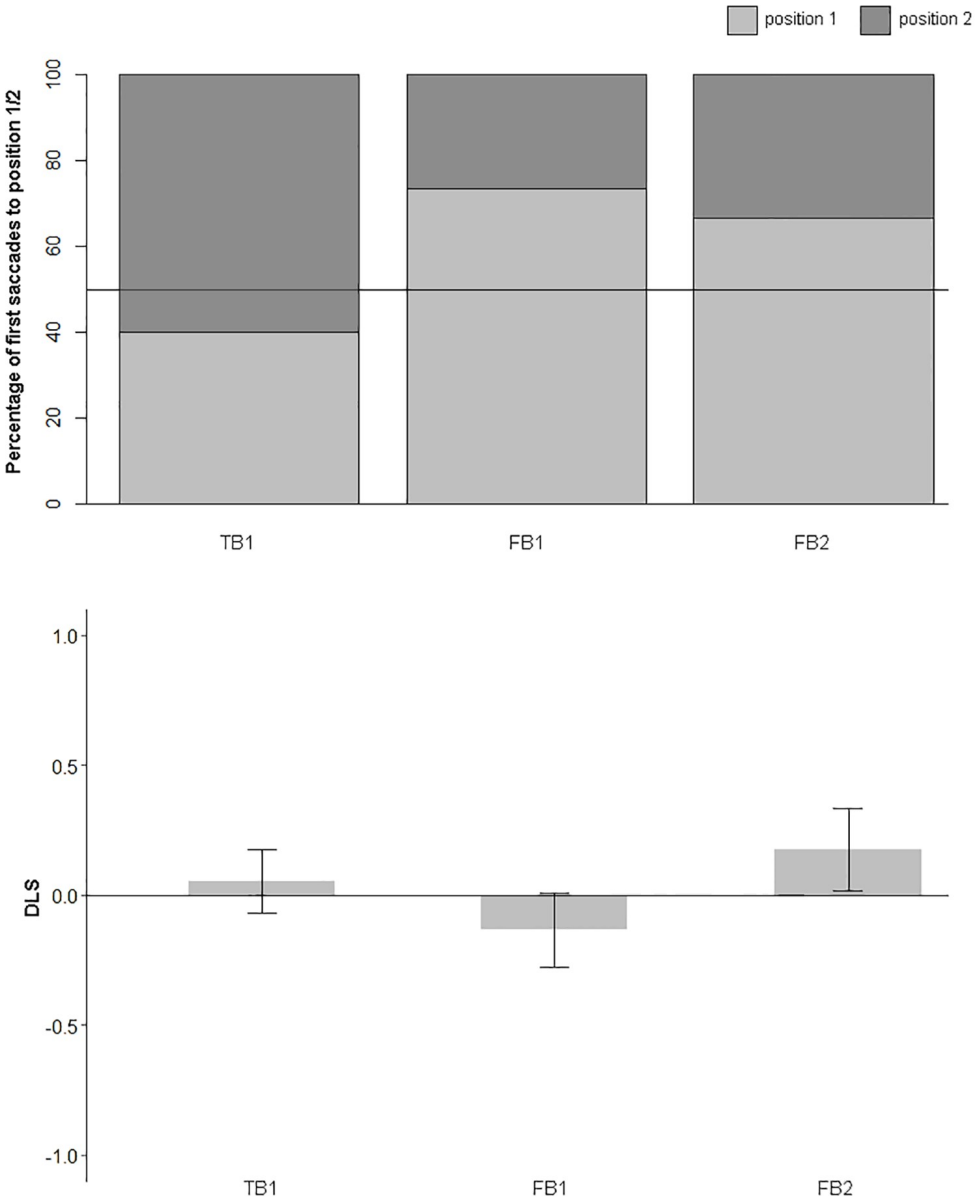
Percentage of first saccades (top) and DLS (bottom) to position 1 and 2 in each condition (FB1, FB2, TB1). Error bars correspond to the standard error.

Full datasets are provided at the OSF, DOI 10.17605/OSF.IO/EF629. Results are displayed in Fig 4.

#### First saccade

Binomial tests showed that the first saccade was not significantly more often directed to either location in the FB1 condition (4 out of 15, i.e. 27% to position 2, *p* = .119, but note *BF*_*10*_ = 1.500, indicating no preference for the last object or belief-congruent location, the same applies to the FB2 condition (12 out of 18, i.e. 67% to position 1, *p* = .238, *BF*_*10*_ = 0.743), indicating no preference for the belief-congruent location, and the TB1 condition (12 out of 20, i.e. 60% to position 2, *p* = .503, *BF*_*10*_ = 0.396), indicating no preference for the last object location. The remaining participants (10 in FB1, 7 in FB2, 5 in TB1) showed no eye-movement to either the box 1 or box 2 location. Fishers exact tests revealed no significant difference in frequency of first saccades towards either location between TB1 and FB1, *p* = .087, OR = 3.95, 95% CI [0.80, 23.54], but note *BF*_*10*_ = 2.530, however there were significantly more saccades towards the belief-congruent location in FB2 compared to FB1, *p* = .037, OR = 5.19, 95% CI [1.00, 33.13], see Fig 4.

#### DLS

One-sample t-tests were used to investigate whether the differential looking score differed from zero in each condition. DLS_belief-based_ did not significantly differ from zero either in FB1 (*M*(25) = -0.134, *SD* = 0.677, 95% CI [-0.41, 0.15]), *t*(24) = -0.987, *p* = .334, *d* = 0.20, *BF*_*10*_ = 0.327, or in FB2 (*M*(25) = 0.178, *SD* = 0.746, 95% CI [-0.13, 0.49]), *t*(24) = 1.195, *p* = .244, *d* = 0.24, *BF*_*10*_ = 0.302. Similarly, the DLS_where-object-last-was_ in the TB1 condition did not differ from zero (*M*(25) = 0.054, *SD* = 0.582, 95% CI [-0.19, 0.29]), *t*(24) = 0.460, *p* = .649, *d* = 0.09, *BF*_*10*_ = 0.232.

An independent samples t-test showed no significant difference of DLS_belief-based_ between FB1 and FB2, *t*(48) = -1.548, *p* = .128, *d* = -0.44, *BF*_*10*_ = 0.606, indicating comparable performance in terms of belief-congruency. The analysis revealed no significant difference of DLS_where-object-last-was_ between FB1 and TB1, *t*(48) = 1.048, *p* = .300, *d* = 0.30, *BF*_*10*_ = 0.344, indicating comparable looking durations towards positions 1 and 2. A linear regression revealed no significant effect of the number of passed familiarization trials on the DLS_belief-based_ in conditions FB1 and FB2, *F*(1, 97) = 0.001, *p* = .974, *BF*_*10*_ = 0.212, see Fig 4.

#### Exclusion rates

Out of overall 157 participants, 82 were excluded, due to missing gaze data (n = 1), experimenter error (n = 1) or not passing the familiarization criterion (n = 80), resulting in a total dropout rate of 52.23% and a dropout rate due to failed familiarization of 51.61%. In the original study by Southgate et al. (2007) 36 participants were tested overall with a total dropout rate of 44.44% and a dropout rate due to failed familiarization of 35.48%. Fishers exact test showed no significant difference between dropout rates of the current and the original study, *p* = .118, OR = 0.52, 95% CI [0.21, 1.22], *BF*_*10*_ = 0.901.

### Discussion Study 3

Study 3 aimed to improve the design by removing confounds and using a more flexible familiarization criterion. Results remain largely inconclusive: the exclusion rates were still very high, despite the changes in familiarization criteria. Concerning anticipatory looking patterns, there was no evidence for belief-tracking, nor for low-level object-tracking. Given that participants showed low levels of anticipation overall, these results remain particular difficult to interpret.

It should be noted that Study 3 did not use an occluder through which the actress reaches (see e.g., [9,10]), but rather used a design in which the boxes lit up to attract participants gaze. However, this manipulation was effective in attracting participants’ anticipatory looks, as 53 out of 75 participants showed a first saccade in the first test trial.

## Overall Discussion

The aim of the current study was to explore why some but not other implicit Theory of Mind tasks could previously be replicated. In particular, we tested whether low-level (sub-mentalizing) attentional processes (tracking the last location of the target object) rather than Theory of Mind (tracking the agent’s belief) could account for previous ambiguous replication patterns of AL FB tasks. To this end, three studies implemented conceptual replications of the Southgate et al. (2007) task with its two conditions FB1 and FB2. In addition, a new control condition (TB1) was devised, that was identical to FB1 with the exception that the protagonist witnessed all events, with the following rationale: If participants still show anticipatory looking in TB1, this indicates that they engage in more low-level processes such as tracking where the object last was rather than belief-ascription.

The main results were the following: First, the original patterns of belief-consistent anticipatory looking (in FB1 and FB2) could largely not be replicated. The only exception was adults’ performance in FB2 in Study 1 which was in line with belief-tracking. This finding, though, is puzzling (given that participants did not reveal analogous anticipation in the much less complex FB1 condition while at the same time showing anticipation in the control condition TB1), and stands in need of clarification. Second, there was some evidence for low-level object-tracking (in the TB1 condition) in Studies 1 and 2, but not in Study 3. Third, attempts to improve familiarization and exclusion procedures in Study 3 were not successful, and the number of successfully passed familiarization trials also did not affect gaze patterns.

All in all, the current set of studies presents a complex and ambiguous pattern of results. No consistent pattern of anticipatory looking could be identified across the three studies, contrasting nativist and two-systems accounts. However, not even the use of low-level sub-mentalizing rules by participants can account for the observed inconsistent pattern. Instead, clear anticipatory looking seems to be absent, questioning the suitability of the method for measuring belief tracking. These results add to a growing body of non- (or partial) replications of anticipatory looking false belief tasks [23,24,28,32,36], while standing in contrast to other recent studies that found positive (albeit modest) belief tracking effects [37,38]. The lack of consistent belief tracking in the present studies, together with many converging negative findings from other recent replication attempts, raise the question whether these tasks are suitable for tapping implicit Theory of Mind. In fact, it should be noted that even in the familiarization trials that were originally designed to check whether participants understand the task, participants showed low rates of correct anticipatory looking. Familiarization trials only require a simple form of goal-directed search. If anticipatory looking does not correctly occur in this simple condition, it is questionable whether the looking behavior really reflects any form of tracking related to the event sequence. This does not answer the question whether implicit Theory of Mind exists, but rather suggests that anticipatory looking may not prove to be a suitable method to study the mentalizing phenomenon or to showcase behavior prediction on the basis of submentalizing. Although other types of tasks, for example tasks investigating alterocentric inference [7,39–41] showed more promising results, they also require further replication.

It may be possible, that the adult participants in the current study were not motivated to follow the videos in the current study, or that the tasks are more suitable for infant populations but not engaging enough for children and adults. Future research (currently ongoing) could therefore aim at creating more engaging videos to increase motivation of participants.

More systematic future research is needed to provide more conclusive answers concerning the suitability and replicability of anticipatory looking tasks for measuring implicit theory of mind or predisposition to submentalize in the face of relevant stimuli. In particular, systematic, preregistered collaborative, multi-lab endeavors with sufficiently large samples sizes are the only way to provide more conclusive answers concerning the suitability and replicability of anticipatory looking tasks for measuring implicit Theory of Mind.

## Supporting information

S1 File Additional preregistered analyses. Additional pre-registered analyes are reported.
(DOCX)
