# Supplementary material for: Why can some implicit Theory of Mind tasks be replicated and others cannot? A test of mentalizing versus submentalizing accounts
Source: PLoS One. 2019 Mar 25;14(3):e0213772. doi: 10.1371/journal.pone.0213772 (PMC6433471; doi:10.1371/journal.pone.0213772)
Supplement: S1 File — Additional pre-registered analyes are reported. (DOCX) [file pone.0213772.s001.docx]

**S1 File. Additional preregistered analyses**

**Study 1 & 2**

**Adults –DLS**

Mixed linear models with subject ID as random effects and Condition as fixed factor showed no significant effect of condition, *F*(2, 72) = 1.321, p = .273, *BF_10_*(Condition+SubjectID) = 0.583.

An independent samples t-test revealed no significant difference between FB2 and TB1, *t*(48) = 0.412, *p* = .682, *d* = 0.119, *BF_10_* = 0.303.

**Children – DLS**

An independent samples t-test revealed no significant difference between FB2 and TB1, *t*(48) = -1.986, *p* = .053, *d* = -0.573, but note *BF_10_* = 1.382.

**Study 3**

**First saccade**

Fishers exact test revealed no significant differences in looking behavior to position 2 compared to 1 between TB1 and FB2, *p* = .745, OR = 0.76, 95% CI [0.16, 3.41], *BF_10_* = 0.404.

**DLS**

An independent samples t-test revealed no significant difference between FB2 and TB1, *t*(48) = -0.659, *p* = .513, *d* = -0.19, *BF_10_* = 0.256.
